# Supplementary material for: Exploring the Pathophysiologic Cascade Leading to Osteoclastogenic Activation in Gaucher Disease Monocytes Generated via CRISPR/Cas9 Technology
Source: Int J Mol Sci. 2023 Jul 7;24(13):11204. doi: 10.3390/ijms241311204 (PMC10342917; doi:10.3390/ijms241311204)
Supplement: Supplementary file 1 [file ijms-24-11204-s001.zip › Supplementary material.pdf]

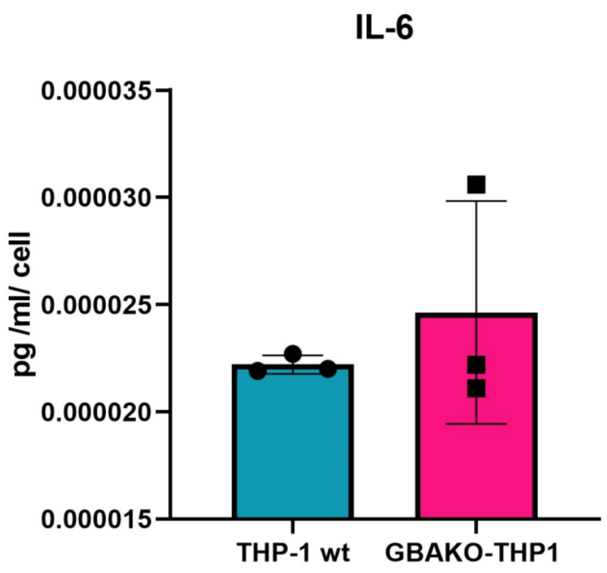

Figure S1 IL-6 levels in the culture supernatant of THP-1 wt and GBAKO-THP1 monocytes. The levels of IL-6 released to the culture media were quantified using a Simple Plex assay (ELLA). Data were normalized via the number of cultured cells and expressed as means ± SD of three independent experiments. There is no statistically significant difference between the groups ($p > 0.05$ t-test).

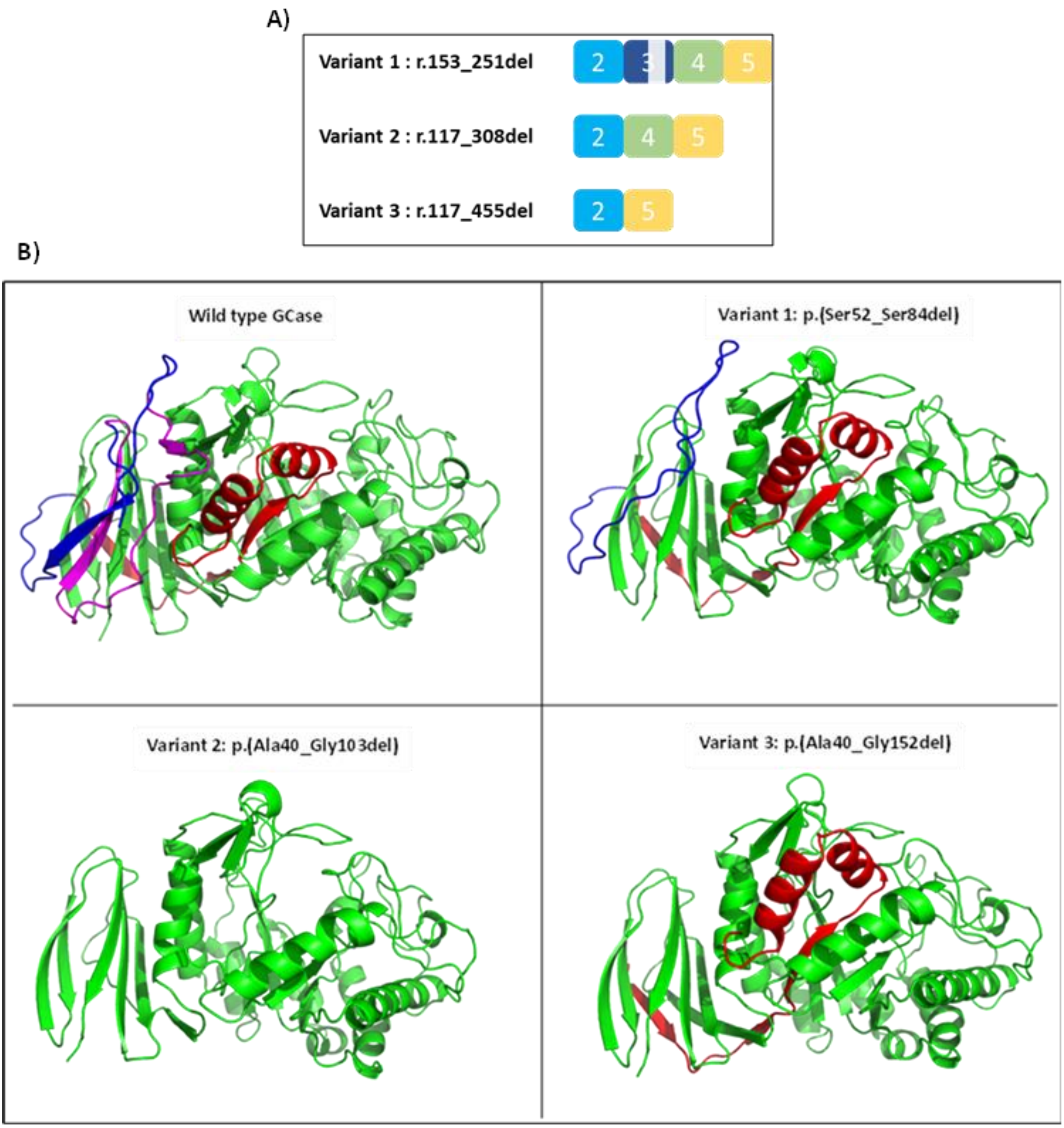

Figure S2 A). Schematic representation of transcripts variants expressed in GBAKO-THP1 cells **B)** Location of deleted GCase residues on the 3D structural model. Wild-type GCase was made using the PDB id:1OGS. The variant's predicted structures were obtained from their amino acid sequences using AlphaFold. All PDB files were loaded in PyMOL for graphical rendering and to highlight the deleted amino acid sequences. Magenta, blue and red fragments show the sequence absent in variant 1, variant 2 and variant 3 respectively.
